# Traitement séquentiel de la lithiase biliaire versus chirurgie seule: analyse par le score de propension

**DOI:** 10.11604/pamj.2013.14.145.2171

**Published:** 2013-04-11

**Authors:** Imane Toughrai, Samir Ahid, Said Ait Laalim, Karim Ibn Majdoub, Khalid Mazaz, Khalid Ait Taleb

**Affiliations:** 1 Service de Chirurgie Viscérale B. CHU Hassan II de Fès, Maroc; 2 Laboratoire de Biostatistique, de Recherche clinique et d'Epidémiologie. Faculté de Médecine et de Pharmacie de Rabat, Maroc; 3 Service de Chirurgie Viscérale A. CHU Hassan II de Fès, Maroc

**Keywords:** Lithiase biliaire, laparoscopie, sphinctérotomie endoscopique, chirurgie, cholelithiasis, laparoscopy, sphincterotomy, surgery

## Abstract

**Introduction:**

L'objectif étant de comparer, rétrospectivement, les résultats du traitement combiné séquentiel à ceux de la chirurgie ouverte chez des patients pris en charge pour lithiase vésiculaire (LV) et lithiase de la voie biliaire principale (LVBP) durant la même hospitalisation

**Méthodes:**

L’étude a concerné 132 patients pris en charge au CHU Hassan de Fès entre Décembre 2003 et Décembre 2011. Nos patients ont été répartis sur deux groupes thérapeutiques. Le premier correspondant aux malades ayant eu une sphincterotomie endoscopique première suivie d'une cholécystectomie par voie cœlioscopique. Le deuxième incluant les malades ayant eu un traitement exclusivement chirurgical. La comparaison des deux groupes a porté sur les résultats en termes de morbi-mortalité, de séjour post opératoire et d'efficacité du traitement.

**Résultats:**

L'appariement des deux groupes thérapeutiques a permis de retenir 64 patients. L’âge moyen a été de 54 ans ± 14 avec un sexe ratio F/H de 2. Quatorze pourcents de nos malades avaient des tares associées. L'ictère ± angiocholite a été noté chez 76,6% de nos patients. Des anomalies du bilan hépatique ont été relevées chez plus de 70% des malades. L’échographie abdominale et le scanner ont été réalisés chez 97% et 30% des malades respectivement. La médiane du séjour post opératoire a été de 2,5 jours pour le premier groupe versus 5 jours pour le deuxième. Nous avons eu un cas de décès post opératoire dans le premier groupe. Les complications post opératoires ont été à type d'un cas de fistule biliaire et 2 cas de sepsis dans le premier groupe. Deux cas de lithiase résiduelle ont été observés dans le groupe de chirurgie seule.

**Conclusion:**

Le choix d'une stratégie de prise en charge de la lithiase biliaire devra prendre en considération le plateau technique disponible et le degré d'expertise des opérateurs. Dans l'attente d'une maîtrise parfaite du traitement de la LVBP par laparoscopie par notre jeune équipe, le traitement combiné séquentiel, nous semble une alternative thérapeutique acceptable.

## Introduction

La lithiase biliaire est une pathologie fréquente dont les complications sont potentiellement graves. Sa prise en charge a beaucoup évolué ces dernières années avec les progrès qu'ont connu l'endoscopie interventionnelle et de la cœliochirurgie. La multiplicité des études contrôlées à ce sujet devra aider au choix d'une stratégie adaptée, en tenant compte du plateau technique et des compétences disponibles.

## Méthodes

Il s'agit d'une étude rétrospective des patients pris en charge, pour lithiase biliaire entre Décembre 2003 et Décembre 2011, aux services de chirurgie et de gastroentérologie du centre hospitalier universitaire de Fès. Nous avons inclu les patients ayant présenté une lithiase vésiculaire (LV) et une lithiase de la voie biliaire principale (LVBP) compliquée ou non d'angiocholite et qui ont bénéficié d'un traitement des deux au cours de la même hospitalisation. Nous avons exclu les patients qui avaient une pancréatite grave et chez qui la cholécystectomie a été réalisée lors d'une hospitalisation ultérieure.

Nous avons colligés 132 patients qu'on a répartis en deux groupes thérapeutiques. Le premier correspond aux malades ayant eu un traitement combiné séquentiel. Celui-ci a comporté une cholangiopancréatographie rétrograde (CPRE) et une sphincterotomie endoscopique préopératoire (SE) suivies d'une cholécystectomie par voie cœlioscopique. Le deuxième groupe est celui des malades qui ont eu un traitement exclusivement chirurgical. Cette dernière approche thérapeutique a été retenue en cas d'indisponibilité ou d’échec de la SE. Nous avons procédé à une étude comparative des deux groupes, portant sur les résultats de notre prise en charge. Les paramètres évalués ont été: la mortalité, la morbidité, la durée du séjour post opératoire et l'efficacité du traitement jugée sur l'apparition ou non de lithiase résiduelle dans les suites lointaines.

L'appariement des deux groupes, moyennant le score de propension, a porté sur: l’âge, les tares associées, la forme clinique, la cholestase et l'existence de pancréatite associée. L'analyse des données a été effectuée sur un logiciel SPSS version 13.0. Pour l’étude descriptive nous avons utilisé les moyennes ± écart-type (ET), les médianes et les pourcentages. La distribution des variables quantitatives a été vérifiée par le test de Kolmogorov-Smirnov. Pour la comparaison des moyennes, on a fait appel au test de Mann Whithney. Pour mesurer l'association entre deux variables qualitatives, nous avons utilisé les tests de Chi 2 et l'exact de Fisher.

## Résultats

Au terme de l’étude, sur un total de 132 malades, l'appariement a permis de retenir 64 patients pour comparer les résultats des deux méthodes thérapeutiques.

L’âge moyen de nos patients a été de 54 ans ± 14 (médiane: 50 ans; extrêmes: 25-80 ans). Il s'agissait de 43 femmes et 21 hommes, soit un sexe ratio F/H de 2. Quatorze pourcent de nos malades avaient des tares associées à type de diabète, hypertension artérielle et cardiopathie. La symptomatologie biliaire a été révélatrice chez tous nos patients. Ainsi, 94% (60/64) d'entre eux ont présenté une colique hépatique. L'ictère et l'angiocholite ont été relevés, respectivement, chez 76,6 (49/64) et 59,4% (38/64) des malades. Une angiocholite grave a été observée chez 11% (7/64) des patients. Une cholécystite et une pancréatite aigüe (PA) œdémateuse ont été, respectivement relevées dans 26,6(17/64) et 14% (9/64) des cas (Tableau 1).

**Tableau 1:** Données sociodémographiques

Données sociodémographiques	%(nb cas)
Age (ans) *	54(±14)
Sexe ratio: F/H	2
Tares associées	14(9/64)
Colique hépatique	94(60/64)
Ictère choléstatique	76,6(49/64)
Angiocholite	59,4(38/64)
Angiocholite grave	11(7/64)
Cholécystite	26,6(17/64)
Pancréatite aigue	14(9/64)

%Valeurs en pourcentage. Nombre de malades entre parenthèses.

* Valeur: moyenne ±ET

Le bilan biologique réalisé à l'admission a objectivé une cholestase et une cytolyse dans 73,4 et 80% des cas respectivement. Une insuffisance rénale, un taux de prothrombine bas et une thrombopénie ont été retrouvés chez tous les patients qui ont présenté une angiocholite grave, soit dans 11% (7/64) des cas. Le Tableau 2 résume l'ensemble des perturbations biologiques chez nos malades.

**Tableau 2:** Perturbations biologiques

Paramètres biologiques	%(nb cas)
GB (/mm3) ≥11000	40,6(26/64)
Hb (g/dl) ≤10	4,7(3/64)
Plaquettes (/mm3) ≤100000	11(7/64)
TP ≤80%	11(7/64)
Urée (g/l) ≥0,5, Créatinine (mg/l) ≥ 14	11(7/64)
GOT(u/l)≥ 46, GPT(u/l) ≥ 46	73,4(47/64)
BT (mg/l) ≥12, GGT (u/l) ≥38, PAL (u/l) ≥ 300	80(51/64)

GB: globules blancs. Hb: hémoglobine.TP: taux de prothrombine. GOT et GPT: transaminases. BT: bilirubine totale. GGT: gamma glutamine transférase. PAL: phosphatases alcalines.%: Valeurs en pourcentages. Nombre de malades entre parenthèses.

L’échographie a permis de poser le diagnostic de lithiase biliaire chez 97% (62/64) des cas. Une tomodensitométrie abdominale a été nécessaire chez 30% (19/64) des malades pour confirmer le diagnostic et stadifier la PA associée. La bili-IRM et l’écho endoscopie ont été respectivement réalisées dans 7,8% et 1,6% des cas.

Tous les malades ont bénéficié des mesures de réanimation adaptées à la gravité du tableau clinique et incluant systématiquement une antibiothérapie à large spectre, des antalgiques et la vitamine K. Les malades qui ont eu une angiocholite grave ont été admis en milieu de réanimation.

Dans le groupe de chirurgie seule, l'abord a été fait par une laparotomie sous costale droite chez tous les malades. Nous avons relevé une cholécystite associée chez 53% des malades de ce groupe (17/32). Les constatations concernant la VBP ont confirmé les données pré opératoires. Le geste a consisté en une cholécystectomie avec extraction de calculs par cholédocotomie. L'intervention s'est terminée par la mise en place d'un drain de Kehr chez 87,5% des malades et une anastomose cholédoco duodénale dans 12,5% des cas. Le drain de Keher a été retiré à J + 15 après contrôle systématique par cholangiographie.

Dans le groupe du traitement combiné, après cholangiographie rétrograde, la sphincterotomie a été systématique. L'extraction de calculs a été faite par des pinces Dormia. La cholécystectomie a été réalisée par voie cœlioscopique 2 à 3 jours plus tard.

La médiane du séjour post opératoire a été de 5 jours dans le groupe de chirurgie seule et de 2,5 jours dans le premier groupe. Nous avons eu un cas de décès post opératoire en rapport avec une angiocholite grave dans le groupe de traitement combiné. Un cas de fistule biliaire et 2 cas de sepsis ont été relevés, également, dans ce groupe. Deux cas de lithiase résiduelle ont été observés dans le groupe de chirurgie seule et traités par SE (Tableau 3).

**Tableau 3:** Comparaison des résultats des deux groupes

Paramètres	Chirurgie seule (n = 32)	Traitement combiné (n = 32)	p
Décès	0	1	0,492
**Complications**			
FB	0	1	0,207
Sepsis	0	2	
Séjour post-op*	5	2,5	0,005
Lithiase résiduelle	2	0	0,497

post-op: post-opératoire. FB: fistule biliaire.

* Valeur: médiane. P < 0,05 est statistiquement significatif.

La comparaison des deux groupes n'a pas retrouvé de différence statistiquement significative concernant le taux des complications et la survenue de lithiase résiduelle. Le séjour post opératoire était significativement moindre dans le groupe du traitement combiné avec une différence de 2 jours et demi (p < 0,05). Nous avons cherché une association entre la durée du séjour post opératoire et l'existence de tares, de cholestase, de pancréatite ainsi que la gravité du tableau clinique. Nous avons conclu à l'absence de différence significative statistiquement entre les deux groupes de malades concernant ces différents paramètres. Le séjour post opératoire moindre dans le groupe du traitement combiné est, donc, uniquement lié à la procédure elle-même (Tableau 4).

**Tableau 4:** Association entre la durée du séjour post opératoire (variable dépendante) et les différentes variables explicatives

Variables explicatives	Odds ratio	IC à 95%	p
Voie d'abord	0,747	0,086-6,512	0,792
Geste chirurgical	2,999	0,893-10,076	0,760
Angiocholite grave	1,528	0,523-4,459	0,438
Pancréatite aigue	6,611	0,544-80,359	0,138
Choléstase	1,527	0,270-8,616	0,127

P < 0,05 est statistiquement significatif.

## Discussion

Le développement incessant des techniques d'endoscopie interventionnelle, a fait que celle-ci prenne de plus en plus d'ampleur dans le traitement de la LVBP. Elle a surtout été au centre de stratégies combinées, associant une CPRE avec SE au traitement chirurgical de LV. D'un autre côté, l’évolution de la cœlioscopie et l'expertise croissante des chirurgiens ont imposé depuis plusieurs années le traitement cœlioscopique de la LV et de la LVBP comme une méthode de choix.

Nombreuses sont les études qui se sont intéressées à ces différentes méthodes, les comparant en terme de faisabilité technique, de succès, de morbi-mortalité, de séjour hospitalier et de coût globale. Le taux de succès rapporté pour le traitement combiné avec SE pré opératoire est de 80% à 95% [1, 2]. Il varie de 92 à 100% pour le traitement combiné en un temps [1–4]. Le traitement « tout » cœlioscopie atteint des taux de succès de 85% à 95% [1, 5, 6]. La mortalité ne dépasse pas 1% pour le traitement cœlioscopique. Elle est quasi nulle pour les autres méthodes [1, 2, 5–8]. Les complications surviennent dans moins de 10% des cas pour les traitements combinés. Ce taux varie de 4 à 17% pour la laparoscopie et il s'agit de complications mineures [1, 5, 7, 9].

Les résultats rapportés dans la littérature laissent suggérer que le traitement combiné séquentiel en un ou deux temps de même que le «tout» laparoscopie sont comparables [10]. Cependant, il persiste beaucoup de divergences entre les équipes. La tendance à adopter ou non l'une ou l'autre approche peut se justifier comme suit:

- Le traitement combiné comporte la morbidité et la mortalité propre de la SE qui sont respectivement de 5 à 11% et de 0,5 à 1,5% [1, 4]
- Les complications liées à la cœlioscopie sont majoritairement mineures contrairement au risque de PA et d'angiocholite encouru lors de la SE [6, 9]
- Le traitement combiné séquentiel a l'inconvénient de faire appel à deux anesthésies et peut aussi allonger la durée globale d'hospitalisation [4]
- La faisabilité du traitement combiné en un temps est tributaire de la disponibilité d'un endoscopiste entrainé au bloc opératoire et surtout d'une très bonne collaboration entre les équipes de chirurgie et d'endoscopie interventionnelle. De plus, l'insufflation gastrique lors du temps endoscopique peut gêner le déroulement de la cholécystectomie. La combinaison des deux méthodes au cours d'une même anesthésie est certes avantageuse, mais se fait au prix d'un allongement du temps de l'intervention [1, 3, 11]
- Le traitement « tout » laparoscopie requiert une bonne maîtrise des techniques de cœlioscopie et une bonne sélection des malades au début de l'expérience [12]. De plus, la durée opératoire peut être allongée de 50 à 100% par rapport à une cholécystectomie standard [7]
- Le séjour hospitalier est incontestablement moindre de même que le coût global de la prise en charge, lorsqu'il s'agit d'un traitement combiné en un temps ou d'une approche purement cœlioscopique [1, 3, 4, 6, 7, 13]

Le traitement séquentiel avec SE pré opératoire continue à avoir la préférence d'un nombre non négligeable de chirurgiens [1, 10, 13]. Nous adhérons totalement à ce point de vue et considérons, par ailleurs, que le traitement combiné en un temps est une alternative intéressante. En effet, elle entre en compétition avec la coelioscopie qui est considérée par beaucoup d'auteurs comme la méthode d'avenir. Dans l'attente d'une bonne maîtrise par notre équipe du traitement totalement cœlioscopie de la lithiase biliaire, le traitement combiné séquentiel nous a permis de diminuer significativement le séjour hospitalier avec des rapports coût/efficacité et efficacité/risque satisfaisants.

## Conclusion

Le choix de l'une ou l'autre stratégie devra prendre en considération, outre la disponibilité d'un plateau technique adéquat, le niveau d'expertise des opérateurs en endoscopie interventionnelle et en cœliochirurgie. Dans l'attente d'une maîtrise parfaite du traitement de la LVBP par laparoscopie par notre jeune équipe, le traitement combiné séquentiel, nous semble une alternative thérapeutique acceptable en termes de séjour post opératoire, de morbidité et d'efficacité.
